# Utility of Feathers for Avian Influenza Virus Detection in Commercial Poultry

**DOI:** 10.3390/pathogens12121425

**Published:** 2023-12-07

**Authors:** Shahan Azeem, Baoqing Guo, Yuko Sato, Phillip C. Gauger, Anna Wolc, Kyoung-Jin Yoon

**Affiliations:** 1 Department of Veterinary Microbiology and Preventive Medicine, College of Veterinary Medicine, Iowa State University, Ames, IA 50011, USA; sazeem@uvas.edu.pk; 2 Institute of Microbiology, Faculty of Veterinary Science, University of Veterinary and Animal Sciences, Lahore 54000, Pakistan; 3 Department of Veterinary Diagnostic and Production Animal Medicine, College of Veterinary Medicine, Iowa State University, Ames, IA 50011, USA; bqguo@iastate.edu (B.G.); ysato@iastate.edu (Y.S.); pcgauger@iastate.edu (P.C.G.); 4 Department of Animal Science, College of Agriculture and Life Sciences, Iowa State University, Ames, IA 50011, USA; awolc@iastate.edu; 5 Hy-Line International, Dallas Center, IA 50063, USA

**Keywords:** avian influenza virus, poultry, feather, real-time polymerase chain reaction, half-life

## Abstract

The present study evaluated the potential utility of feather samples for the convenient and accurate detection of avian influenza virus (AIV) in commercial poultry. Feather samples were obtained from AIV-negative commercial layer facilities in Iowa, USA. The feathers were spiked with various concentrations (10^6^ to 10^0^) of a low pathogenic strain of H5N2 AIV using a nebulizing device and were evaluated for the detection of viral RNA using a real-time RT-PCR assay immediately or after incubation at −20, 4, 22, or 37 °C for 24, 48, or 72 h. Likewise, cell culture medium samples with and without the virus were prepared and used for comparison. In the spiked feathers, the PCR reliably (i.e., 100% probability of detection) detected AIV RNA in eluates from samples sprayed with 10^3^ EID_50_/mL or more of the virus. Based on half-life estimates, the feathers performed better than the corresponding media samples (*p* < 0.05), particularly when the samples were stored at 22 or 37 °C. In conclusion, feather samples can be routinely collected from a poultry barn as a non-invasive alternative to blood or oropharyngeal–cloacal swab samples for monitoring AIV.

## 1. Introduction

Avian influenza virus (AIV), a member of the *Orthomyxoviridae* family, is a highly contagious RNA-containing virus that is widespread in aquatic migratory wild birds. During their seasonal migrations, these birds occasionally transmit the virus to domestic poultry [1,2,3]. The AIVs are generally maintained as low pathogenic AIV (LPAIV) strains in migratory waterfowl, and may cause mild disease in naïve poultry [4,5]. Highly pathogenic avian influenza virus (HPAIV) can evolve from LPAIV strains and cause high mortality ranging from 90 to 100% in gallinaceous poultry [6]. Additionally, HPAIV outbreaks in the US between 2021 and 2023 demonstrated that migratory waterfowl could transmit the virus directly to poultry without any adaptation from LPAIV to HPAIV. Epidemics resulting from HPAIV infections can spread rapidly through poultry-producing regions and cause devastating socioeconomic consequences in the poultry industry.

Unprecedented outbreaks of HPAI (highly pathogenic avian influenza) due to H5N2 AIVs of Eurasian–North American lineage occurred in the United States in 2014–2015 [7]. These outbreaks occurred with suspected interaction between migratory waterfowl and poultry and devastated the US poultry industry, especially in the Midwestern states of Iowa and Minnesota. Overall, the epidemic affected 21 states and led to the loss of over 50 million poultry. The economic losses due to the epidemic were estimated at USD 5 billion [8]. In Iowa alone, the H5N2 epidemic affected over 31 million poultry from 77 affected sites representing 18 counties, with the economic losses estimated at USD 1.2 billion [9,10]. More recently, H7N3 and H5N1 HPAIVs of migratory waterfowl origin have been reported in poultry and aquatic mammals, respectively [11,12]. These HPAI epidemics raise questions about the effectiveness of AIV surveillance programs for US domestic poultry.

The National Poultry Improvement Plan (NPIP), a voluntary and cooperative federal–state–industry certification program, was established to monitor selected poultry diseases of economic significance to the US poultry industry and reduce the impact of such disease outbreaks [13]. Under NPIP 9 CRF Chapter 146, participating commercial table-egg layer flocks must be monitored for H5/H7 AIV through routine surveillance programs via approved tests. The USDA-approved enzyme-linked immunosorbent assay (ELISA) or agar gel immunodiffusion (AGID) test is used for serologic monitoring of AIV antibodies. ELISA-positive samples are required to undergo further confirmatory testing via the AGID test within 48 h. AGID-positive samples are further evaluated by a federal reference laboratory using hemagglutination-inhibition (HI) and neuraminidase-inhibition (NI) assays [14].

Additionally, naturally occurring flock mortality or clinically ill birds demonstrating unexplained respiratory disease and decreased egg production must be examined and tested at a diagnostic laboratory for AIV using an approved antigen detection test and serology [14]. In the case of commercial layers, an investigation for AI may be triggered when flocks have mortality exceeding three times the daily average or a five percent drop in egg production for three consecutive days [15]. Antigen-capture immunoassay-non-negative oropharyngeal or cloacal (OPC) samples must be further tested using a real-time reverse transcription-polymerase chain reaction (rtRT-PCR) or virus isolation (VI), as per the protocol recommended by National Veterinary Services Laboratories (NVSL). All PCR- or VI-non-negative OPC samples are further tested and confirmed by a federal reference laboratory (i.e., NVSL).

However, the NPIP testing scheme (i.e., bird sample-based testing) has shortfalls and may not provide diagnostic information for the early detection of an AIV incursion in commercial poultry. Both serology and antigen detection tests require a small subset (11 samples in the case of commercial table egg layers) irrespective of the flock size. The frequency of sampling has large gaps, wherein long-lived birds like commercial layers are only tested three times: once prior to a pullet flock moving to a lay site, once every 12-month period, and once within 21 days prior to movement to disposal. LPAIVs do not readily produce overt clinical signs of disease or death, complicating AIV detection via active surveillance. The delayed detection of LPAIVs circulating in poultry populations is a risk factor for the emergence of HPAIVs, since H5 and H7 LPAIVs are known to mutate into HPAIVs [16,17].

In addition to bird samples, other sample matrices have been evaluated for AIV surveillance or monitoring. Various environmental samples and reservoirs, such as drinking water, drinker biofilm, feces, cages, egg belts, house floor, manure belts, manure pits, and air, have shown their utility for AIV detection in poultry barns [18,19,20,21,22,23,24]. While the environmental samples are easy to collect, these samples commonly require clarification and/or virus concentration before testing. It may be challenging to interpret positive test results when samples are collected after cleaning and disinfection and are tested using a nucleic acid-based test.

For migratory wild birds, fresh fecal samples (droppings), feathers, and lake sediment have been evaluated as an alternative to cloacal swabs to surveil the birds for AIV [18,25,26,27]. Among these, feathers can also be easily obtained from commercial poultry facilities due to molting or regular feather turn-over, preening, and feather pecking [28,29,30]. Feathers may serve as an alternative surveillance sample to detect AIV in commercial poultry, knowing AIV is shed in oronasal secretions besides feces [31], and such bodily fluids can frequently be in contact with the beak and the feathers via normal bird behaviors such as bill- or nose-wiping, preening, and grooming [32,33,34]. The detection of HPAIV has been reported in feathers plucked from live birds, particularly in their calami, as the virus can replicate in pulp [26,35]. Hence, feathers are likely to be of diagnostic value for a prolonged period compared to other environmental samples [35]. Despite these potential advantages of feather samples, very limited feather sampling has been applied to commercial poultry for AIV detection [35,36]. The following laboratory study was conducted to evaluate the potential utility of fallen feathers as an alternative environmental sample for the accurate, non-invasive, and convenient detection of AIV in commercial poultry houses (i.e., population testing).

## 2. Materials and Methods

### 2.1. Study Design

A randomized complete block design was used to evaluate feathers as an alternative sample matrix. Feather samples (mostly contour feathers) were collected from commercial layer facilities in Iowa with no history of AIV. The feather samples were weighed into 10 g aliquots (approximately ten feathers) and placed in individual Ziploc^®^ bags (Chicago, IL, USA). Serial 10-fold dilutions of an H5N2 LPAIV isolate, which was obtained from a migratory waterfowl during the USDA wild bird surveillance program for HPAIV, were made in Dulbecco’s modified Eagle media (DMEM) and spiked onto each 10 g feather sample to obtain the desired virus concentration, ranging from 10^6^ to 10^0^ median embryo infective dose (EID_50_) per gram, along with negative controls (i.e., spiked with virus-free DMEM). For virus spiking, a nebulizing device (LMA MAD Nasal Intranasal Mucosal Atomization Device, Teleflex, Morrisville, NC, USA) was used to spray the virus onto the feathers. The procedure was performed in a laminar flow biosafety cabinet. The virus-spiked DMEM was prepared similarly by making 10-fold serial dilutions of the virus in DMEM and stored at the same temperature for the specified time to serve as a positive control. Three replicates of feathers for each virus concentration were randomly assigned to one of the three incubation times (24, 48, and 72 h) and four different incubation temperatures (−20, 4, 22, and 37 °C). The times and temperatures were selected to mimic routine submission conditions of samples from commercial poultry facilities to a diagnostic laboratory for testing. In total, 288 aliquots (=8 virus concentrations × 3 aliquots × 3 times × 4 temperatures) were prepared for evaluation. After each specified incubation time at each given temperature, a triplicate of feather samples was picked and stored at −80 °C until analysis via the rtRT-PCR. In addition, 24 feather samples were randomly selected immediately after aliquoting and stored in a −80 °C freezer, serving as controls without the effect of time or temperature, referred to as 0 h samples.

### 2.2. Sample Preparation for Testing

Viruses on feathers were eluted in DMEM. To achieve that, 10 mL of cold DMEM was added to a 15 mL conical tube containing one gram of spiked feathers. The tubes were placed on an orbital shaker set at 200 rpm for 1 h at ambient temperature (23.0–23.5 °C) and centrifuged at 200× *g* for 10 min at 4 °C. Eluates were then collected into tubes; supplemented with the final concentration of 2000 IU/mL penicillin G, 0.2 mg/mL of streptomycin sulfate, 0.25 mg/mL gentamicin sulfate, and 500 IU of amphotericin B; and stored at −80 °C until viral RNA extraction.

### 2.3. RNA Extraction

Viral RNA was extracted from each feather eluate via magnetic bead-based separation technology using an Ambion^®^ MagMAX^TM^-96 Viral RNA Isolation Kit (Life Technologies, Carlsbad, CA, USA) following the protocol provided by the manufacturer. Per the manufacturer’s instructions, the procedure was performed in a KingFisher^®^ 96 automated magnetic particle processer (ThermoFisher Scientific, Prussia, PA, USA). Extracted viral RNA was eluted in a 100 µL elution buffer.

### 2.4. Real-Time Reverse Transcription-Polymerase Chain Reaction

A commercial one-step real-time multiplex RT-PCR kit (VetMAX™-Gold AIV Detection Kit; Life Technologies, Austin, TX, USA), designed to target viral matrix (M) and nucleoprotein genes, was used to detect influenza viral RNA in feather samples [37]. The PCR reaction was set up in a 25 µL volume containing 12.5 µL of 2× multiplex RT-PCR buffer, 1.0 µL nuclease-free water, 1.0 µL of influenza virus primer/probe mix, 2.5 µL of multiplex RT-PCR enzyme mix, and 8.0 µL of RNA template or control. Xeno™ RNA Control, supplied with the kit, was included in the reaction as an internal control for RNA purity to assess possible PCR inhibition from samples. The Influenza Virus-Xeno™ RNA Control (1000 copies/µL) included in the kit was used as a positive amplification control (PAC). Nuclease-free water was used as a negative amplification control. Thermocycling was performed in a 7500 Fast PCR System (Applied Biosystems, Foster City, CA, USA) under the following conditions: reverse transcription at 48 °C for 10 min, reverse transcriptase inactivation/initial denaturation at 95 °C for 10 min, and 40 cycles of amplification and extension (95 °C for 15 s and 60 °C for 45 s). The PCR data were analyzed per the manufacturer’s recommendations using the Manual Cycle Threshold (Ct) setting and default baseline cycle 3–15. The AB AIV master detector threshold was determined by multiplying the delta Rn of PAC at cycle 40 by 0.05. Amplification plots were reviewed to ensure positive controls crossed the threshold and negative controls did not. AIV RNA and Xeno™ RNA controls were detected by using FAM and VIC dyes, respectively. Samples with Ct > 38 were considered negative, as per the manufacturer’s guidelines. The PCR data were used to obtain virus titer estimates based on a standard curve generated by quantitatively performing rtRT-PCR on serial 10-fold dilutions of the virus with known titer (EID_50_/mL) and plotting the Ct value of each dilution against the expected viral titer of the corresponding dilution.

### 2.5. Data Analysis

The qualitative results (i.e., positive and negative) of the PCR on the samples were compared to determine the limit of detection (LOD) of the PCR for AIV in feathers under different temperature conditions. The lowest viral titer spiked into feathers that resulted in 100% detection in the three replicates of eluates was considered LOD in a conservatively estimated manner [38].

To estimate the half-life of viral RNA in the feather and media samples, log_10_-transformed EID_50_ values were analyzed with a linear model using the “lm” function in R software version 3.3.3 [39]. The values were increased by one to enable log transformation for negative samples. A covariate of log_10_-transformed initial viral dose [log_10_(InitialConcentration + 1)] was fitted to adjust for the starting level of viral load and the fixed effect of sample type to account for differences in mean viral titers between DMEM and feathers. A covariate of time (0, 24, 48, and 72 h) was fitted to estimate the decline rate of the virus level. The interaction between sample types and the decline rates of viral titer was estimated to determine differences between the feathers and the DMEM. The half-life (*t*_1/2_) of H5N2 LPAIV in the DMEM and the feathers was determined separately at each temperature [40,41]. Briefly, the half-life was determined by fitting a simple linear regression model accounting for the initial viral concentration. Once the regression equation was constructed, the estimate of the slope and standard error were used to obtain a point estimate of the half-life and its confidence interval, as previously reported [40]. If the slope was non-negative or was not significantly different from zero, the upper bound of the half-life confidence interval was incalculable and interpreted as infinity. The estimate of AIV’s *t*_1/2_ in feather samples was compared to that in DMEM, which was considered the ideal storage material, to assess the effect of feather samples on virus detection.

## 3. Results

PCR-based estimates of viral titer in each feather sample immediately after spiking (i.e., 0 h) were very close to those anticipated based on the viral titer in the original cell culture material measured via PCR-based estimates and dilution factor during processing (Table 1). The PCR reliably detected AIV in feather samples when spiked with the virus to yield a viral titer of 10^3^ EID_50_/mL or higher, regardless of storage temperature (Table 2).

Based on half-life estimates of the spiked LPAIV, the virus stability was better in feathers than in DMEM (*p* < 0.05; Table 3). The shortest half-life estimate of 63.34 h was observed when the virus was stored at 37 °C in the media; at the same temperature, the half-life of the virus in the feathers was longer than in the media. When the spiked feather and media samples were compared across temperatures, there was a substantial difference in the half-life of AIV between the feather and media samples incubated at 37 °C (F-value = 34.01; *p*-value < 0.05) and 22 °C (F-value = 10.38; *p*-value < 0.05). However, such a difference was not observed at lower temperatures (4 and −20 °C). At all temperatures for feathers, and 22, 4, and −20 °C for media, relatively longer half-life estimates, or a slower decline rate of virus titers, were observed. Since the decline rate was not significantly different from zero, no meaningful half-life estimates could be obtained (Table 3).

## 4. Discussion

In the present study, we evaluated the potential use of feathers as an alternative environmental sample type for the early detection and surveillance of AIV in domestic poultry facilities. Feather samples were consistently positive for AIV after PCR when spiked with 10^3^ EID_50_/mL or a higher titer of the virus, regardless of storage temperatures (Table 2). It should be noted that the elution process before PCR testing further dilutes the virus on feathers 1:10 or more. A previous study reported that feathers from H5N1 HPAVI-positive wild ducks contained more than 10^4^ EID_50_/mL of the virus [42]. Moreover, in chickens, LAPIVs are typically shed in respiratory secretions at a titer of approximately 10^2.5^ to 10^4.0^ EID_50_/mL [43]. Therefore, the observed level of detection indicates that feathers from an AIV-exposed poultry barn may be sufficient for PCR-based monitoring for AIV. If necessary, pooling more feathers to elute can be an option to increase the virus titer in an eluate for testing.

The half-life assessment demonstrated no decay of the virus amount on the spiked feathers under study conditions. In particular, no detrimental effect on the viral RNA amount on the spiked feathers was observed while being stored at room temperature or higher for three days (Table 3), suggesting that feather samples can be diagnostically advantageous for sample storage and shipping to a laboratory for AIV PCR testing when sampling areas are geographically remote from the lab. It should be noted that the half-life can be different if virus-spiked feathers are kept longer than 72 h, as natural bodily fluids (e.g., oronasal secretions and feces) are expected to have organic materials that can positively or negatively impact the survivability or stability of viruses or viral genetic material on feathers.

It is not known if these PCR-positive feathers contained infectious viruses, since the virus isolation test was not performed. However, other investigators have reported that viable AIV was detected in feathers collected from experimentally infected ducks for longer than in oropharyngeal/tracheal and cloacal swabs [44]. Higher viral loads of HAPIV (2.3.4.4b) have also been reported in feathers compared to tracheal and cloacal swabs [45]. The better survivability of infectious AIV in feathers was also demonstrated compared to drinking water and feces when stored at 4 °C and 20 °C [42]. Therefore, a biosafety precaution must be taken when handling potentially virus-laden feathers.

For AIV testing in chickens, flight or contour feathers are preferred [36], although immature pectorosternal feathers can also be collected from live birds as they pose minimum discomfort when plucked. Within a feather, the calamus, which is part of the shaft held in the feather follicle, is known to be the best site for HPAIV detection since viable viruses have frequently been isolated from the calamus of freshly plucked feathers, and the calamus has shown to harbor the virus [46]. However, the present study used eluates from whole feathers sprayed with LPAIV for testing because the study simulated the situation where birds could have AIV from oronasal secretions and fecal matter on their feathers due to their normal behaviors and interactions. In addition, we found the feather shaft and calamus difficult to process using a scissor and a tissue homogenizer or paddle blender due to the firmness of the feather shaft, which can be a challenge to technical staff at diagnostic laboratories. Furthermore, plucking feathers from a large number of live birds in a commercial poultry operation can be an animal welfare issue, not to mention labor demand. The use of shed or fallen feathers can be an excellent alternative to plucking, as it offers a non-invasive and convenient sample collection for population testing.

The utility of shed or fallen feathers for AIV monitoring is supported, as the present study demonstrated that feathers could be a good reservoir of AIV once the virus is on the feathers. Such virus-laden loose feathers should be available in an AIV-positive poultry house, as the virus is shed in bodily fluids [47]. Nevertheless, field-based studies using feathers collected from commercial poultry houses are desired to confirm the usefulness of feather sampling and to develop sampling guidelines, such as the frequency of collection and number of feathers to collect, for reliable AIV monitoring or surveillance in the natural setting. The study observation and the same idea may be extrapolated for monitoring other poultry respiratory viral pathogens, although it remains to be further studied in the field.

## Figures and Tables

**Table 1:** PCR-based estimates of virus titer (median egg infective dose, log EID_50_/mL) in feather eluates and media immediately after spiking with an H5N2 low pathogenic avian influenza virus (AIV) at various concentrations.

Sample	Expected AIV Titer (EID_50_/mL) in Virus-Spiked Sample					
	10^6^	10^5^	10^4^	10^3^	10^2^	10^1^
Feather eluate ^A^	4.51 ^B^	3.29	2.46	1.05	0	0
Dulbecco’s modified Eagle’s media	6.21	4.96	3.76	2.72	1.65	0.48

^A^ Viruses on a feather (approximately 1 g) were eluted into 10 mL Dulbecco’s modified Eagle’s media by immersing the spiked feather into the media in a tube and gently vortexing on an orbital shaker for 1 h at ambient temperature. ^B^ AIV titer (EID_50_/mL in log_10_) was estimated using a quantitative real-time reverse transcription-polymerase chain reaction assay specific for matrix and nucleoprotein genes of the virus.

**Table 2:** Detection of H5N2 low pathogenic avian influenza virus (AIV) spiked in feather and Dulbecco’s modified Eagle’s media (DMEM) after 24, 48, and 72 h storage at various temperatures by real-time reverse transcription-polymerase chain reaction.

Temperature (°C)	Matrix	AIV Titer (EID_50_/mL or EID_50_/g) Expected in Each of the Virus-Spiked Samples													
		0 h							24 h						
		10^6^	10^5^	10^4^	10^3^	10^2^	10^1^	10^0^	10^6^	10^5^	10^4^	10^3^	10^2^	10^1^	10^0^
−20	DMEM	3/3 ^A^	3/3	3/3	3/3	3/3	1/3	0/3	3/3	3/3	3/3	3/3	3/3	1/3	0/3
4									3/3	3/3	3/3	3/3	3/3	0/3	1/3
22									3/3	3/3	3/3	3/3	3/3	1/3	0/3
37									3/3	3/3	3/3	3/3	0/3	0/3	0/3
−20	Feather eluates ^B^	3/3	3/3	3/3	3/3	0/3	0/3	0/3	3/3	3/3	3/3	3/3	1/3	1/3	0/3
4									3/3	3/3	3/3	3/3	2/3	0/3	0/3
22									3/3	3/3	3/3	3/3	2/3	0/3	0/3
37									3/3	3/3	3/3	3/3	2/3	1/3	0/3
		**48 h**							**72 h**						
		**10^6^**	**10^5^**	**10^4^**	**10^3^**	**10^2^**	**10^1^**	**10^0^**	**10^6^**	**10^5^**	**10^4^**	**10^3^**	**10^2^**	**10^1^**	**10^0^**
−20	DMEM	3/3	3/3	2/3	3/3	3/3	2/3	0/3	3/3	3/3	3/3	3/3	3/3	1/3	0/3
4		3/3	3/3	3/3	3/3	3/3	2/3	0/3	3/3	3/3	3/3	3/3	3/3	0/3	1/3
22		3/3	3/3	3/3	3/3	3/3	1/3	0/3	3/3	3/3	3/3	3/3	3/3	1/3	0/3
37		3/3	3/3	3/3	3/3	2/3	1/3	0/3	3/3	3/3	3/3	3/3	0/3	0/3	0/3
−20	Feather eluates	3/3	2/3	2/3	3/3	0/3	0/3	0/3	3/3	3/3	3/3	3/3	0/3	1/3	0/3
4		3/3	3/3	3/3	1/3	2/3	0/3	0/3	3/3	3/3	3/3	3/3	0/3	0/3	0/3
22		3/3	3/3	3/3	3/3	2/3	1/3	1/3	3/3	3/3	3/3	3/3	2/3	0/3	1/3
37		3/3	3/3	3/3	3/3	1/3	1/3	0/3	3/3	3/3	3/3	3/3	2/3	0/3	0/3

^A^ Number of positive samples/Number of tested samples. The grey shed represents the limit of detection at 100% probability of detection (3/3). ^B^ Viruses on a feather (approximately 1 g) were eluted into 10 mL Dulbecco’s modified Eagle’s media by immersing the spiked feather into the media in a tube and gently vortexing on an orbital shaker for 1 h at ambient temperature.

**Table 3:** The half-life of H5N2 low pathogenic avian influenza virus (AIV) spiked into feather samples and Dulbecco’s modified Eagle’s media (DMEM) at various temperatures as estimated using a real-time reverse transcription-polymerase chain reaction (rtRT-PCR).

Sample Matrix	Incubation Temperatures (°C)			
	37	22	4	−20
DMEM	63.34 ^A^ (46.60, 98.83) ^B^	∞ ^C^	∞	∞
Feather eluate	∞	∞	∞	∞

^A^ The feather or media was spiked with various concentrations (10^0^ to 10^6^ EID_50_/mL) of AIV and tested for virus titer via rtRT–PCR immediately and every 24 h while incubating at various temperatures for 72 h. ^B^ The 95% confidence interval of the estimated half-life. ^C^ The slope was not significantly different from zero; hence, the upper bound of the confidence interval is incalculable and is interpreted as infinity.

## Data Availability

Data can be obtained by contacting the corresponding author. No public link to data is available.
